# Predicting Effects of Ocean Acidification and Warming on Algae Lacking Carbon Concentrating Mechanisms

**DOI:** 10.1371/journal.pone.0132806

**Published:** 2015-07-14

**Authors:** Janet E. Kübler, Steven R. Dudgeon

**Affiliations:** Department of Biology, California State University, Northridge, California, United States of America; University of California, Merced, UNITED STATES

## Abstract

Seaweeds that lack carbon-concentrating mechanisms are potentially inorganic carbon-limited under current air equilibrium conditions. To estimate effects of increased atmospheric carbon dioxide concentration and ocean acidification on photosynthetic rates, we modeled rates of photosynthesis in response to *p*CO_2_, temperature, and their interaction under limiting and saturating photon flux densities. We synthesized the available data for photosynthetic responses of red seaweeds lacking carbon-concentrating mechanisms to light and temperature. The model was parameterized with published data and known carbonate system dynamics. The model predicts that direction and magnitude of response to *p*CO_2_ and temperature, depend on photon flux density. At sub-saturating light intensities, photosynthetic rates are predicted to be low and respond positively to increasing *p*CO_2_, and negatively to increasing temperature. Consequently, *p*CO_2_ and temperature are predicted to interact antagonistically to influence photosynthetic rates at low PFD. The model predicts that *p*CO_2_ will have a much larger effect than temperature at sub-saturating light intensities. However, photosynthetic rates under low light will not increase proportionately as *p*CO_2_ in seawater continues to rise. In the range of light saturation (I_k_), both CO_2_ and temperature have positive effects on photosynthetic rate and correspondingly strong predicted synergistic effects. At saturating light intensities, the response of photosynthetic rates to increasing *p*CO_2_ approaches linearity, but the model also predicts increased importance of thermal over *p*CO_2_ effects, with effects acting additively. Increasing boundary layer thickness decreased the effect of added *p*CO_2_ and, for very thick boundary layers, overwhelmed the effect of temperature on photosynthetic rates. The maximum photosynthetic rates of strictly CO_2_-using algae are low, so even large percentage increases in rates with climate change will not contribute much to changing primary production in the habitats where they commonly live.

## Introduction

Continued absorption of anthropogenic emissions of CO_2_ from burning fossil fuels, into seawater will inevitably lead to further declines in oceanic pH with predictable consequences for oceanic chemistry [1,2]. Changing ocean chemistry is expected to shift ratios of resources as well as environmental conditions, thereby favoring some groups of organisms at the expense of others [3–6]. Much attention has focused on calcifying organisms that are predicted to be vulnerable to ocean acidification as a result of lower saturation states of calcium carbonate species as reviewed in [7–9]. Non-calcifying phototrophs (e.g., macroalgae and seagrasses) are predicted to benefit in terms of growth, if not always in terms of photosynthetic rate, from ocean acidification (OA) due to the enhanced availability of dissolved CO_2_ in the ocean.

In contrast to studies of OA on putatively vulnerable taxa, there has been less effort directed towards questions about the magnitudes of the effect on putative beneficiaries of OA. There are many important questions about these organisms in need of resolution to better predict the consequences of climate change to oceanic ecosystems, such as, “will, and if so, by how much will algal productivity be enhanced in acidified coastal water?”, “will productivity be determined by changing *p*CO_2_ or temperature?”, or “how will the mode of inorganic carbon acquisition interact with other determinants of productivity as *p*CO_2_ increases?”.

Recent comprehensive meta-analyses [6,7,10] reviewing the effects of OA on marine organisms, highlight, the paucity of investigation relative to other groups, and interesting patterns of response of non-calcifying algae. Studies of fleshy macroalgae thus far, generally show significantly enhanced growth under OA, yet without significant enhancement of photosynthesis in response to moderately elevated *p*CO_2_. The effect of sampling few fleshy macroalgae coupled with inattention to the species’ mechanisms of inorganic carbon uptake may mask variation present in the magnitude of effects OA has on macroalgal physiology, productivity and growth.

Whereas all green and brown macroalgae use both HCO_3_^-^ and CO_2_ sources of inorganic carbon for photosynthesis, not all red algae can take up HCO_3_^-^ directly. Approximately 35% of all red algal species tested use only dissolved CO_2_ for photosynthesis [4,11]. These species are characteristic of light-limited environments such as beneath overhanging rock walls in intertidal or subtidal habitats, especially in temperate and sub-arctic environments [12,13]. Non bicarbonate using red algae are found in very high abundance (> 60% of species) in low light environments around Tasmania, Australia [14]. Demand for dissolved inorganic carbon is less when PFD is low.

Photosynthesis of CO_2_-using red macroalgae is potentially carbon-limited at present oceanic dissolved *p*CO_2_ and is a hypothesized link to their occurrence in low light habitats [15,16]. Therefore, a straightforward prediction of the effects of ocean acidification is an increase in productivity and growth of strict CO_2_-using algae. Greater inorganic carbon availability could lead to ranges extending into new, higher light intensity, habitats. A prior study of the CO_2_-using red alga, *Lomentaria articulata*, showed enhanced growth with increased *p*CO_2_ [16] supporting that prediction, but there are few other well studied CO_2_-using marine red algae. Critical gaps remain with respect to predicting the effects of climate change on macroalgal production. Despite extensive knowledge of the effects of temperature on macroalgal photosynthesis [17–20] the combined effects of increasing *p*CO_2_ and temperature are less well known. Macroalgae with CCMs tend to have positive additive or synergistic effects of temperature and OA [21, 22]. A key question arising from projections of climate change is whether the known responses to temperature change and to increased inorganic carbon supply are additive or synergistic. Light supply also interacts with both temperature [20] and inorganic carbon uptake [23, 24]. We expected light supply to interact with climate change in algal productivity, possibly modulating the interaction between OA and temperature effects.

Our ability to predict the effects of climate change on algal productivity and growth are contingent upon our understanding of how changing environments affect algal physiological responses. In this context, we have developed a model specific to CO_2_-using red macroalgae. The model predicts effects of increased *p*CO_2_ (OA) and temperature on photosynthetic production at different light intensities. Comparing theoretical predictions with observations, as more data becomes available, will provide a test of synergies in the combined effects of light intensity with increasing acidification and temperature in the oceans on productivity of CO_2_-using algae. The model is parameterized using known temperature-dependent diffusivities of the different inorganic carbon species in seawater of given characteristics (salinity, pressure, viscosity, pH, total alkalinity), and photosynthetic responses of CO_2_-using marine red macroalgae to variations in light and temperature.

This model represents a starting point for choosing the combinations of multiple environmental factors and corresponding habitats that may be of special interest as ocean acidification continues. The parameterizing studies were primarily done as single stressor dose response studies rather than multi-stressor factorial manipulations and we are able to leverage areas of overlap. Our synthesis of the available data allows for the detection of habitats where physiological and ecological studies can be focused to maximize efficiency of further work. For example, if ocean acidification will alleviate other limitations to the range of the macrophytes in question, where can we predict their ranges to expand?

## Materials and Methods

### The Model

Our model of photosynthetic production uses the Hill-Whittingham (1955) equation, which is the appropriate model for aquatic systems where diffusive uptake of inorganic carbon can be limiting [25–27]. For red macroalgae that take up only CO_2_, this is the case regardless of the presence of external carbonic anhydrase (CA) in the cell wall. CA only affects the rate of equilibration between CO_2_ and HCO_3_^-^ [28] and ultimately CO_2_ crosses the plasmalemma by diffusion.

Photosynthetic rate is modeled as a hyperbolic function of CO_2_ concentration and permeability in the following equation:

$$\;\;\;\;V_{o}=0.5*\left\{\left(P_{u}*K_{\frac{1}{2}}+P_{u}*S+V_{\text{max}}\right)-{\left[{\left(P_{u}*K_{\frac{1}{2}}+P_{u}+V_{\text{max}}\right)}^{2}-\left(4*P_{u}*S*V_{\text{max}}\right)\right]}^{\frac{1}{2}}\right\}$$ (1)

where;

*V*_0_ = Net photosynthetic rate in μmol C-fixed · m^-2^ · s^-1^

*P*_*u*_ = permeability coefficient in m · s^-1^; the ratio of the temperature-dependent diffusion of CO_2_ and pathlength from outside the cell to the plastid

*K*_1/2_ = half-saturation constant of photosynthesis at specified conditions of light and temperature; in μmol · m^-3^

*S* = Substrate concentration in μmol · m^-3^; the dynamic flux expressed as the difference in dissolved CO_2_ outside (C_ext_) and inside (C_int_) the cell

*V*_*max*_ = Maximum net photosynthetic rate at specified conditions of light and temperature in μmol C-fixed · m^-2^ · s^-1^

Data obtained from the model reflect acclimation to steady state conditions of light, temperature and pH/carbon balance. We assume no adaptive evolution on the timescale of the model.

### Model Parameterization

Parameters of this equation are influenced by light availability, temperature, hydrodynamics and/or nutrient concentration. Some relationships are the direct effect of water chemistry, for example, the positive effect of temperature on diffusion rate. Other parameters, such as the CO_2_ concentration gradient between the seawater medium and the location of carbon fixation, depend on boundary layer characteristics and P_max_ under the prevailing conditions. Thus we have a basis to explore the potentially interactive effects of light, CO_2_ availability and temperature on algal productivity, through the Hill-Whittingham equation.

The half-saturation constant (*K*_1/2_) of photosynthesis of strict CO_2_-using red algae is light-dependent [12]. We assumed a linear dependence of *K*_1/2_ on light intensity because the functional relationship of the light-dependence of *K*_1/2_ between sub-saturating and saturating light intensities has not been resolved.

It is well established that photosynthetic rates of macroalgae are affected by temperature and the availabilities of light and dissolved inorganic carbon (DIC) [18,23,29,30]. We used the averages of photosynthetic rate parameters (per unit thallus area) at both sub-saturating and saturating light intensities and temperatures ranging from 5 to 30°C from published studies of known CO_2_-using red macroalgal species (e.g., genera including *Lomentaria*, *Delesseria*, *Plocamium*) to parameterize photosynthetic performance (i.e., *V*_max_) of the model [11,12,18], [see Supporting Information, S1 Table for details]. The mass-specific rates of photosynthesis of *Lomentaria* species reported in [18] were converted to area-specific rates using the biomass to area conversion factor for *Lomentaria* provided in [12].

We modeled a linear increase of photosynthesis (due to presumed declining photorespiration) of 10% between the lowest modeled *p*CO_2_ and the highest modeled *p*CO_2_ (reduced photorespiration). At normoxia and *p*CO_2_ of approximately 380 μatm in the gas phase, photosynthesis of CO_2_-using red macroalgae is reduced by 5–6% by photorespiration [31–35]. As increasing CO_2_ at constant *p*O_2_ will reduce the mole fraction ratio of O_2_:CO_2_ at the site of carbon fixation by Ribulose-1,5-bisphosphate-carboxylase (RUBISCO), OA is expected to reduce photorespiratory losses of CO_2_-using red algae.

The flux of dissolved CO_2_ supplied to the site of RUBISCO is itself influenced by temperature both directly and indirectly. The direct effects of temperature on CO_2_ flux include its solubility and diffusivity in seawater [36,37]. At a given seawater pH, *p*CO_2_ decreases, but diffusivity of CO_2_ increases, with increasing temperature. These two parameters govern the external concentration of dissolved CO_2_ supplied to a cell on the thallus surface. Values for the concentrations and diffusivities of DIC species at 5°C intervals from 5 to 30°C were provided by CO_2_Calc [36] and [37], respectively. Constants for use in calculating carbonate parameters were those of [38] based on the total pH scale. The formulation by [39] was used for the dissociation of KHSO_4_ to estimate carbonate parameters.

An indirect effect of temperature on CO_2_ flux is internal CO_2_ dynamics caused by temperature-dependent rates of metabolism [12]. The boundary conditions for internal CO_2_ concentration were established in the following way. Maximum internal CO_2_ was assumed to occur in darkness and was taken as the value in equilibrium with CO_2_ in external seawater. Minimum internal CO_2_ was assumed to occur under light saturation and was taken as the steady-state equilibrium corresponding to the respiration rate at a given temperature. At sub-saturating light intensities, a linear relationship of C_int_-depletion with increasing light intensity was modeled between these boundary conditions corresponding to the linear rate of carbon-fixation by photosynthesis in the light-limited portion of the curve. Together, these direct and indirect temperature-dependent effects determine the flux to, and concentration of, CO_2_ substrate for RUBISCO.

In addition to modeling photosynthetic rate, we estimated values for isotopic composition of carbon (δ^13^C) and discrimination against ^13^C (Δ) as an indicator of the physiological state of photosynthetic metabolism in response to OA and warming. At each seawater pH simulated, the mole fractions of dissolved CO_2_ and HCO_3_^-^/CO_3_^2-^ of total inorganic carbon were calculated. These values were multiplied by the carbon isotopic fractionation of dissolved CO_2_ with respect to dissolved HCO_3_^-^ in seawater at each temperature calculated using the temperature-dependent fractionation parameters for the carbonate system provided in [40]. These calculated values served as the isotopic composition value of the source seawater for calculating predicted values of δ^13^C of the plant using the formula [41]:

$$\delta^{13}C_{org}=\frac{\delta^{13}C_{CO_{2}}-\alpha_{org}+1}{\alpha_{org}}$$ (2)

where, *δ* ^13^ *C*_org_ = carbon isotopic composition of the plant organic biomass

*δ* ^13^ *CO*_2_ = carbon isotopic composition of dissolved CO_2_ in seawater of a given temperature

α_org_ = overall fractionation of^13^C in dissolved CO_2_

We modeled the overall fractionation of ^13^C in dissolved CO_2_ (α_org_) due to diffusion in seawater (α_d_ = 1.0007) and carboxylation by RUBISCO (α_c_ = 1.029) following the analysis by [41]:

$$\alpha_{org}=\alpha_{d}*\left(\frac{\left(C_{b}-C_{c}\right)}{C_{b}}\right)+\alpha_{c}*\left(\frac{C_{c}}{C_{b}}\right)$$ (3)

where, *C*_*b*_ = [CO_2_] in the bulk medium,

*C*_*c*_ = [CO_2_] internally at the site of RUBISCO

These carbon availability parameters reflect the weighting of fractionation due to diffusion in seawater and the activity of RUBISCO in the overall effect of the fractionation parameter. Possible values of α_org_ range from 1.0007 (diffusion limits any internal accumulation of CO_2_ precluding discrimination by RUBISCO) to 1.029 (fractionation solely due to discrimination by RUBISCO). We model these relative weights as a function of light availability (Table 1). Support for the chosen values is provided by data of [15] showing that in low-light CO_2_-using red algae, 80–85% of the limitation of the rate of photosynthesis can be attributed to carboxylation. Under light-limitation, ample CO_2_ is available and, thus, RUBISCO carboxylation primarily determines α_org_. Under high light, diffusion of CO_2_ may limit photosynthesis and fractionation is increasingly influenced by the component due to diffusion.

**Table 1:** Weighting coefficients of fractionation of ^13^C dissolved in CO_2_ due to diffusion (*w*_αd_) in seawater and carboxylation by RUBISCO (*w*_αc_) as a function of light intensity (PPFD).

PPFD	*w*_αd_	*w*_αc_
10	0.02	0.98
35	0.07	0.93
50	0.01	0.90
75	0.15	0.85
100	0.20	0.80
400	0.30	0.70

PPFD measured in μmol photons m^-2^ s^-1^

The discrimination against ^13^C (Δ) was estimated for each CO_2_, temperature and light level simulated from the formula in [41] using the light-dependent weightings of fractionation above to estimate α_org_:

$$\Delta =\frac{\delta^{13}C_{CO_{2}}-\delta^{13}C_{org}}{1+\delta^{13}C_{org}}=\alpha_{org}-1$$ (4)

Comparisons among Δ values obtained from future experiments with those of the modeled curve will elucidate whether those differences could be attributable to carbon-limitation at a given *p*CO_2_, temperature and light intensity.

### Model Evaluation

The model varies parameters related to photosynthetic characteristics (e.g., *K*_1/2_ and *V*_max_) and environmental conditions that determine dissolved CO_2_ concentration and photosynthetic capacity. Our focus was on modeling the individual and combined effects of OA and increased temperature on photosynthetic rates and how this relationship changes with light intensity. Photosynthetic performance was modeled for nine seawater pH values (7.73, 7.76, 7.80, 7.85, 7.88, 7.91, 7.95, 8.07 and 8.10) at each of six different seawater temperatures (5, 10, 15, 20, 25, 30°C). Photosynthetic rate in each of these 54 combinations of seawater pH and temperature was estimated in simulations at six light intensities (10, 35, 50, 75, 100, 400 μmol photons · m^-2^ · s^-1^).

Other environmental and photosynthesis model parameters were held constant across all simulations. The physical and chemical seawater properties input as constants in CO_2_Calc assumed a shallow near surface habitat characteristic of CO_2_-using red macroalgae: salinity = 32‰; pressure = 20 dbars; and Total Alkalinity (TA) = 2200 μmol/kg seawater. We also assumed a well-mixed flow environment with the minimum expected pathlength of 20 μm for diffusive uptake of dissolved CO_2_ [15,42– 44]. A minimal diffusive boundary layer for model results is consistent with all results taken from the literature, which were measured under well-mixed conditions. We additionally explored the effect of increasing boundary layer thickness over a order of magnitude, from 20 through 200 μm, on the principal model outcomes (described in S1 Appendix).

### Analysis of Model Simulations

Modeled rates of photosynthesis at each *p*CO_2_ and temperature combination were analyzed using polynomial regression, which was most appropriate because of the well-known curvilinear response of the photosynthesis-temperature relationship over the range of simulated temperatures [17]. A separate regression analysis was done at each simulated light intensity. The aim of the regressions was to estimate standardized regression coefficients for linear and quadratic terms for CO_2_ and temperature and their interaction. These standardized coefficients were used to infer the relative magnitudes of effect on photosynthetic production caused by variation in CO_2_ and temperature. A total of 13 models that varied in the number and combination of predictor variables (i.e., presence or absence of quadratic and/or linear combinations of predictor variables) were evaluated at each light intensity. The best fit models at each light level were determined from corrected AIC values and their respective weights and relative likelihoods computed. Standardized regression coefficients were estimated by model averaging with unconditional standard errors obtained from the weights of all 13 models using the AICcmodavg (version 1.35, [45]) package in R [46]. The model averaging and multi-model inference approach of [47] employed here is described in S2 Appendix.

## Results

**Fig 1:**
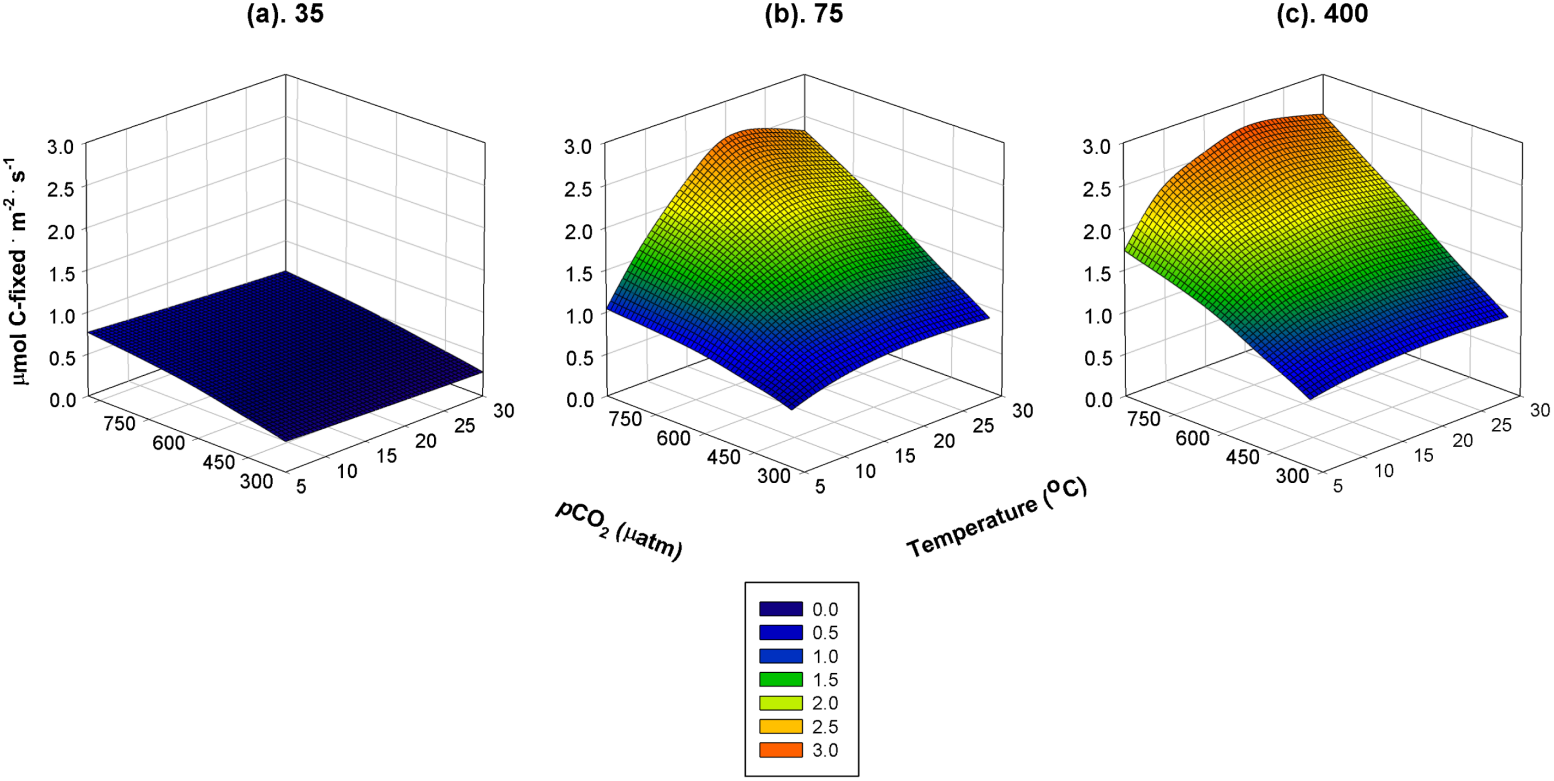
Modeled rates of net photosynthesis as functions of predicted response to *p*CO_2_ and known response to temperature at sub-saturating (35μmol photons · m^-2^ · s^-1^), approximately Ik (75 μmol photons · m^-2^ · s^-1^), and saturating (400 μmol photons · m^-2^ · s^-1^) saturating photon flux densities. Response surface fit using polynomial regression.

**Fig 2:**
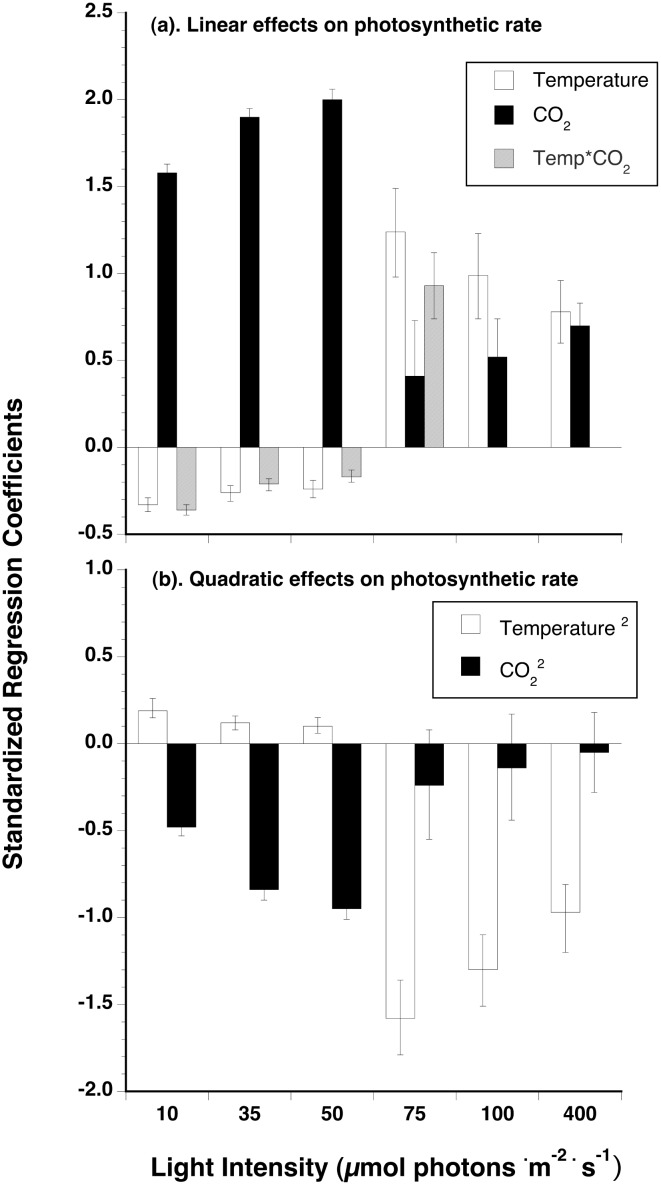
Standardized partial regression coefficients ± standard errors for (A) linear and (B) quadratic components of the best-fit regression model using AICc. Coefficients for temperature (open columns), CO_2_ (filled columns), and the temperature*CO_2_ interaction (stippled columns) are plotted for each of six light intensities ranging from 10 to 400 μmol photons · m^-2^ · s^-1^.

Modeled rates of photosynthesis varied in response to changing *p*CO_2_, changing temperature, and with the interaction of the two. The direction and magnitude of the predicted response to CO_2_ and temperature, however, changed with light intensity (Fig 1). At sub-saturating light intensities, photosynthetic rates are predicted to be low and respond positively to increasing *p*CO_2_, and negatively to increasing temperature (Fig 1A). Consequently, CO_2_ and temperature interacted antagonistically to influence photosynthetic rates. Standardized regression coefficients at low light intensities predict that CO_2_ should have a much larger effect than temperature in both linear and quadratic components (Fig 2). Photosynthetic rates should be most responsive to *p*CO_2_ under sub-saturating light among all of the combinations of light, temperature and CO_2_ simulated. The large (in absolute value) predicted negative quadratic effect of CO_2_ under low light reflects the expectation that photosynthetic rates will not increase proportionately as *p*CO_2_ in seawater continues to rise (Fig 2B). This negative quadratic term approaches 0, and the linear coefficient increases, for CO_2_ as light intensity increases to saturating values indicating a reduced curvature and greater linearity of the response of photosynthetic rates to increasing *p*CO_2_ (cf. CO_2_ in Fig 2A and 2B).

In the vicinity of I_k_ (here, ~75 μmol photons · m^-2^ · s^-1^), the model predicts a switch in the relative importance of CO_2_ and temperature on photosynthetic rates. Linear and quadratic standardized coefficients for CO_2_ decline to absolute values <0.5, whereas those for temperature increase in absolute magnitude to ≥ 1.25 (Fig 2). CO_2_ and temperature coincide in their effect on photosynthetic rates (positive linear and negative quadratic components) and this interaction results in strong predicted synergistic effects on rates especially at high *p*CO_2_ and moderate temperatures (Fig 1B, i.e., 15–25°C). At *p*CO_2_ >700 uatm, photosynthetic rates are expected to double and be markedly responsive to temperature showing the characteristic, asymmetrical, hump-shaped curve with a maximum at 15–20°C. At historical and current *p*CO_2_, the sensitivity to temperature of photosynthetic rates of CO_2_-using macroalgae is less.

A similar magnitude of increased photosynthetic rate with increased *p*CO_2_ and temperature is expected under light saturation with two important differences (Fig 1C). Under light saturation, the effects of CO_2_ and temperature on photosynthetic rate are expected to be additive (i.e., no interaction between CO_2_ and temperature; Fig 2A). Also, modeled rates of photosynthesis of CO_2_-using macroalgae at high light intensities are less sensitive to temperature at high *p*CO_2_ (i.e., negative quadratic coefficient of temperature approaches 0) than are algae near I_k_ (cf. Fig 1B and 1C). Overall under light saturation, the positive responses of photosynthetic rate to increasing CO_2_ or temperature are of equivalent magnitudes with respect to the linear component. The negative quadratic component for CO_2_ is small rendering a largely linear increase of photosynthetic rate to increasing CO_2_, whereas the more negative quadratic coefficient of temperature causes a more curvilinear response (Figs 1C and 2B).

**Fig 3:**
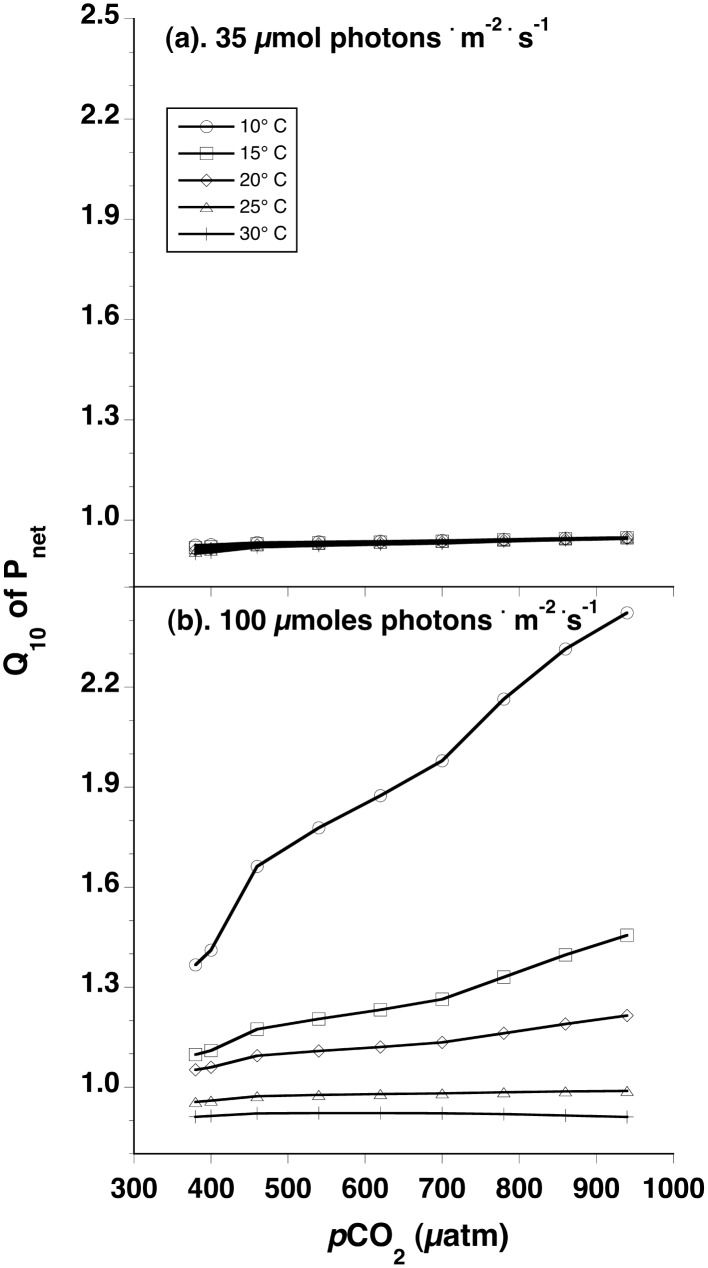
Modeled Q_10_ response of net photosynthesis as a function *p*CO_2_ (μatm) for seaweeds in seawater at 10° (circles), 15° (squares), 20° (diamonds), 25° (triangles), or 30°C (pluses) and representative sub-saturating (35 μmol photons · m^-2^ · s^-1^, top plot), or saturating (100 μmol photons · m^-2^ · s^-1^, bottom plot) photon flux densities.

Fig 3 illustrates how different combinations of temperature and *p*CO_2_ in seawater at representative sub-saturating and saturating light intensities in which macroalgae are grown are predicted to affect Q_10_ responses. Estimated values of Q_10_ at sub-saturating light intensities vary little from ~0.9 at all *p*CO_2_ and temperature levels simulated. In contrast, at light intensities approximating I_k_ or clearly saturating PFD, Q_10_ values exceed 1 at simulated temperatures ≤20°C, increase with increasing *p*CO_2_, especially at 10°C in which Q_10_ estimates exceed 2. At 25 and 30°C, Q_10_ values are approximately 0.9–1.0.

**Fig 4:**
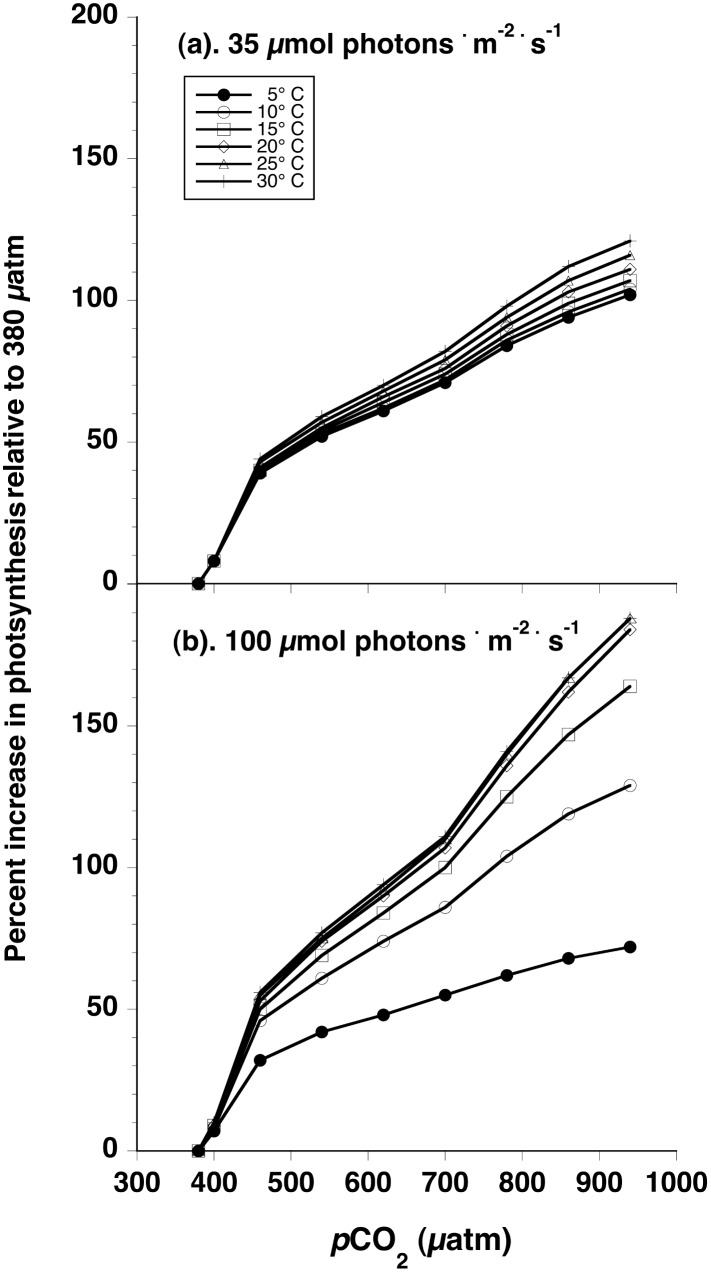
Percentage increase in modeled rates of net photosynthesis, relative to rates at 380 μatm, as a function *p*CO_2_ (μatm) for seaweeds in 5° (filled circles), 10° (open circles), 15° (squares), 20° (diamonds), 25° (triangles), or 30°C (pluses) seawater at representative sub-saturating (35 μmol photons · m^-2^ · s^-1^, top plot), or saturating (100 μmol photons · m^-2^ · s^-1^, bottom plot) light intensities.

Rates simulated under different light, *p*CO_2_ and temperature regimes afford estimates of the percentage increase in photosynthesis relative to the recent past, *p*CO_2_ standard of 380 ppm. At all temperature and light intensities simulated, the greatest percentage increase of photosynthetic rates of up to 50% in response to increasing *p*CO_2_ is predicted to occur between 380 and 460 ppm CO_2_ (Fig 4). Thereafter, further increases in rates to 100% (i.e., doubling photosynthetic rate relative to 380 ppm) are predicted to occur at ~700–800 ppm *p*CO_2_ with algae at warmer temperatures and higher light intensities reaching this level of production at lower *p*CO_2_.

**Fig 5:**
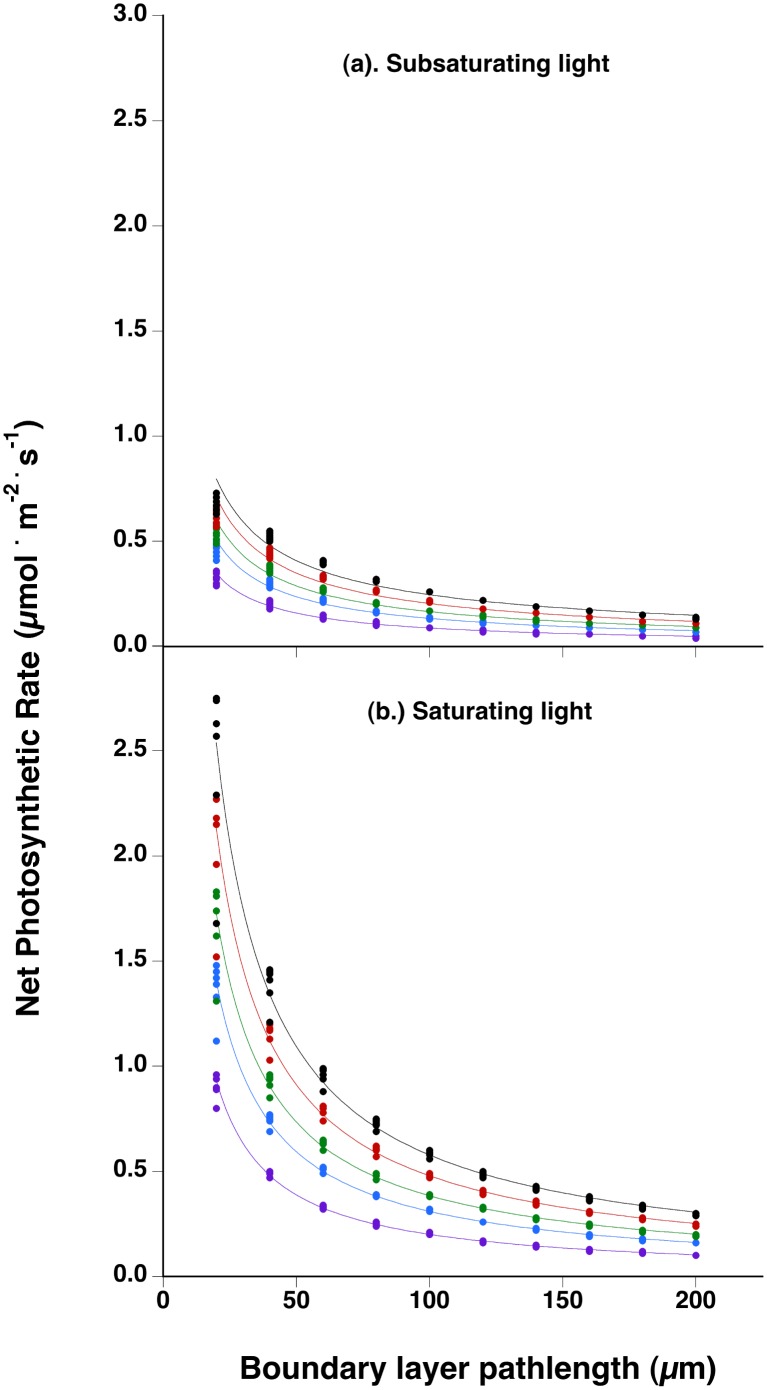
Predicted rates of net photosynthesis as a function of pathlength through the diffusive boundary layer at the thallus surface for (a.) subsaturating light (35 μmol photons · m^-2^ · s^-1^), or (b.) saturating light (400 μmol photons · m^-2^ · s^-1^) intensities. Lines represent power curves fit to predicted points at each combination of temperature (5 to 30°C at 5°C intervals) and *p*CO_2_; purple, 380; blue, 460; green, 620; red, 780; black, 940 μatm)

All of the results presented in Figs 1–4 assume a minimal thickness of the diffusive pathlength through the boundary layer. Fig 5 shows the predicted effect on net photosynthetic rate of increased diffusive pathlength through the boundary layer for plants at different combinations of *p*CO_2_ and temperature at either saturating (400 μmol photons · m^-2^ · s^-1^), or subsaturating (35 μmol photons · m^-2^ · s^-1^) light intensity. Net photosynthetic rates are expected to decline sharply with a doubling of boundary layer from the thinnest pathlength (20 to 40 μm length). Thereafter, declines in photosynthesis continue with increasing pathlength and, in fact, the relationship between net photosynthetic rate and boundary layer thickness across light intensities is described by a negative power law (see S1 Appendix). Two additional predictions are noteworthy with respect to ocean acidification and warming. First, as expected, the higher *p*CO_2_ concentrations in lower pH waters under ocean acidification partially compensates for greater DIC diffusion limitation associated with thicker boundary layers (S1 Appendix). Second, the effect of temperature, especially at, or above, saturating light intensities on photosynthetic rates predicted for minimal boundary layer thickness is progressively eliminated with increasingly thick boundary layers (Fig 5).

### Using discrimination against ^13^C to indicate physiological response to added pCO_2_

**Fig 6:**
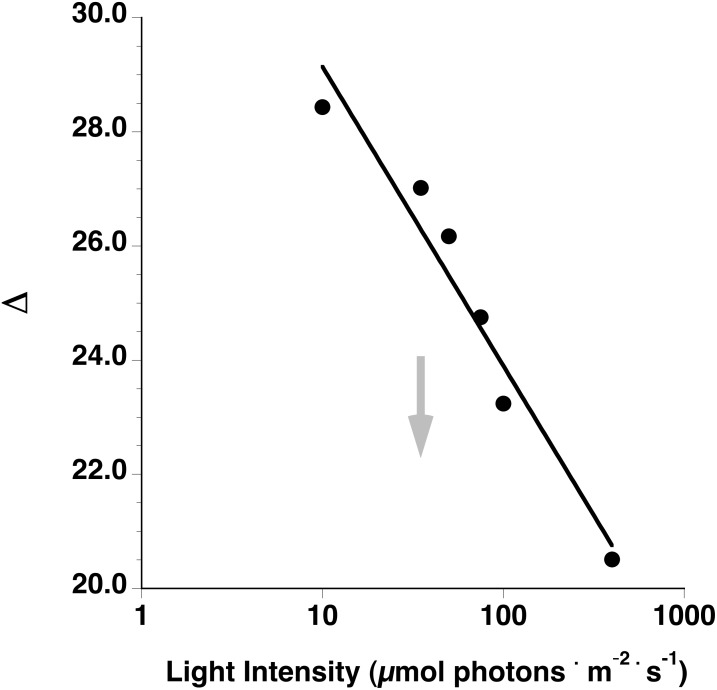
Discrimination against ^13^C by a model CO_2_-using red alga as a function of light intensity. Solid line represents maximum discrimination expected at a given light intensity as a result of a minimal diffusive boundary layer. Increased diffusion path in a thicker boundary layer shifts the discrimination curve down (gray arrow). Note: X-axis scale is logarithmic.

Discrimination against ^13^C by an alga (Δ) occurs with respect to the processes of diffusion of CO_2_ and carboxylation by RUBISCO differently fractionating ^13^C. The line in Fig 6 represents expected values of Δ under well-mixed, minimal boundary layer conditions. Values of Δ are predicted to be inversely related to light intensity (i.e., less discrimination with increasing light intensity; Fig 6). Additionally, as the thickness of the boundary layer increases, so too does the weight of fractionation against ^13^C due to diffusion. This causes a downward shift of the curve of Δ versus light intensity (indicated by gray arrow in Fig 6). Thus, the line expressing the relationship between Δ and light intensity represents the upper boundary (maximal) discrimination expected. Isotopic composition in tissues (δ^13^C) is a function of both isotopic composition of the source seawater and overall fractionation of ^13^C by the alga. Corresponding expected δ^13^C values as a function of seawater temperature and CO_2_ as a fraction of total Carbon are shown in S1 Data.

## Discussion

Concern about effects of climate change on marine life has spawned a rapidly increasing number of studies characterizing responses of algae to increasing CO_2_ and temperature in the oceans. Few of those studies include evaluation of the mechanism of inorganic carbon acquisition operating as interface between the organism and the external carbonate chemistry. Haphazard choices of fleshy algal species as experimental subjects in some cases, or the special concern about calcareous algae in other cases, and the experimental conditions to which they are subjected make an emergent synthesis difficult. Our motivation was to develop a quantitative model to predict the effects of increased CO_2_ on photosynthesis of strictly CO_2_ using (i.e., non-bicarbonate using) red macroalgae, which are estimated to comprise ~35% of all rhodophytes [11] and are over represented among macroalgae communities in deepwater, low transparency water and caves [14]. A second model representing some bicarbonate-using species is in a forthcoming manuscript. We restricted our model to seawater salinities but it could be extended to include the strictly CO_2_ using red algae in fresh water (e.g., *Lemanea spp*.; [42–44, 48]). The CO_2_-user model is based on the known temperature and pH-dependent fractionation of carbon species in seawater [35], dynamics of diffusive uptake of CO_2_ in aquatic plants [25–27], and prior data on photosynthetic responses of CO_2_-using red seaweeds to temperature and light [12,15,16,18]. Such a model provides a tool to assess understanding of the relationships of photosynthesis to key environmental parameters, a valid framework upon which to base predictions of changes in primary production, distribution and abundance, and a basis from the bottom-up to inform hypotheses about community-level implications to climate change.

Analysis of these model outcomes generates predictions that address four fundamental, unresolved questions about productivity of CO_2_-using algae in a changing climate: (1) How much (if any) will productivity be enhanced in acidified coastal waters?, (2) Will increased CO_2_ or temperature be the principal driver of changing algal productivity?, (3) Will changing CO_2_ and temperature act synergistically to influence algal productivity?, (4) How will light supply affect (if at all) photosynthetic responses to increasing CO_2_ and temperature?

### *p*CO_2_ has a stronger effect on photosynthetic rates at subsaturating photon flux densities while temperature had a larger effect on photosynthetic rates under higher light.

The most striking result is the expectation that the individual and interactive effects of CO_2_ and temperature on photosynthetic rates vary with light intensity. CO_2_ has a stronger effect on photosynthetic rate at sub-saturating light intensities and temperature has a stronger effect at, or above, saturating light intensities. Standardized regression coefficients show that the magnitude of the CO_2_ effect on photosynthetic rates is ≥5-fold the effect of temperature when photon flux density is limiting. In addition, under limiting PFD the effects of changing *p*CO_2_ and temperature on photosynthetic rate are predicted to be antagonistic, which leads to a moderate negative interactive effect. At, and above, light saturation, temperature has a positive effect on net photosynthetic rates that is stronger than the positive effect of CO_2_. Whereas they act synergistically in the vicinity of I_k_, their effects on photosynthesis are additive well above I_k_. Further evidence of the light-dependent effects of temperature are suggested by Q_10_ values at each *p*CO_2_ under sub-saturating or saturating light intensities. The Q_10_ of photosynthetic rate is approximately 1 at all *p*CO_2_ levels and temperatures under sub-saturating light, but > 1 and increasing at higher *p*CO_2_ levels from 10–20°C under saturating light.

### OA is predicted to increase photosynthetic rate in algae using dissolved CO_2_ as their inorganic carbon source. The magnitude of the benefit will be largest at warmer temperatures, greater photon flux densities, and high flow (thin boundary layer) conditions.

It is frequently suggested that non-calcifying algae will benefit from ocean acidification, but these suggestions are made without regard to the source of carbon a macroalga uses for photosynthesis, nor the magnitude of the presumed effect. In regard to the former, there is likely to be little direct benefit to bicarbonate-using seaweeds, which are typically saturated for DIC at present [28] and several empirical studies corroborate this (e.g., [49,50]; see also reviews by [6,7]), though there may be indirect benefits. As all algae use dissolved CO_2_ for some fraction of their inorganic carbon supply, the predicted benefits may apply to that fraction of the net primary productivity in those species, as well. Based on our analysis, OA should benefit macroalgae that lack carbon-concentrating mechanisms. The magnitude of this benefit to enhanced productivity *in situ* appears to depend on both light supply and, especially, temperature in the environments inhabited by these algae. The predicted increases in photosynthetic rates are similar across all light environments simulated up to 460–500 μatm *p*CO_2_. The environmental range over which the greatest percentage (50%) benefit of OA is expected is from the current, approximate *p*CO_2_ of 400 to 460 μatm. At higher *p*CO_2_, smaller proportionate increases in photosynthetic rate are predicted especially at lower PFD and temperature. At sub-saturating light intensities, a doubling of photosynthetic rate (relative to that measured at 380 ppm CO_2_) is predicted at ≥800 μatm *p*CO_2_. By contrast, algae living under saturating light should show a doubling of current photosynthetic rates at *p*CO_2_’s ranging from ~650, at temperatures ≥20°C, to ≥800 μatm at 5°C.

### Stimulation of photosynthesis by OA effects on diffusive CO_2_ uptake are not predicted to be a large part of total coastal primary productivity.

These large percentage increases in net photosynthetic rate, are not likely to cause large absolute changes in primary productivity. Maximum rates of net photosynthesis of red algae lacking CCMs are on the order of 1 μmol C-fixed · m^-2^ · s^-1^ at 380 μatm *p*CO_2_. For seaweeds living in habitats typically saturated with light, photosynthetic rates may be expected to reach 2 μmol C-fixed · m^-2^ · s^-1^ at *p*CO_2_’s greater than 700 μatm. For seaweeds living in light-limited habitats, photosynthetic rates may be expected to reach 0.75–1.0 μmol C-fixed · m^-2^ · s^-1^. All marine photorophs supply some part of their inorganic carbon demand through the diffusive uptake of dissolved CO_2_. For most macroalgae, that is small fraction of their primary production. To the extend that additional dissolved CO_2_ is increased there may be marginally great net photosynthetic rate if the CCM does not already completely saturate inorganic carbon supply.

### OA is unlikely to increase total productivity of algae lacking CCMs in their current habitats but may allow for range expansions into brighter habitats

These predicted effects on photosynthesis in response to climate change have implications for the production, distribution and abundance of CO_2_-using rhodophytes and the communities in which they live. Rhodophytes lacking CCMs tend to live in temperate, low-light, cold water environments [13–16], precisely the kinds of conditions predicted to have little enhancement of productivity. Thus, the majority of CO_2_-using, non-calcifying macroalgae are expected have little potential to increase ecosystem-level production in their current, subtidal habitats.

We hypothesize that increased CO_2_ may facilitate range expansion of CO_2_-using algae into new habitats. The potential increase in photosynthetic rate in bright, warmer environments due to alleviation of CO_2_ limitation, predicted by the model, is consistent with this hypothesis. Nevertheless, increased production of CO_2_-using algae with continued rise of *p*CO_2_ in seawater is not without constraint. Our estimates of Δ predict an inverse relationship with light intensity, which is supported by empirical data from three rhodophyte species [30], but even under saturating light (e.g., 400 μmol C-fixed · m^-2^ · s^-1^) discrimination values >20 are predicted. Discrimination against ^13^C and isotopic composition values of CO_2_-using rhodophytes are greatly influenced by the relative weights of fractionation of ^13^C due to diffusion of CO_2_ through the boundary layer and carboxylation by RUBISCO [41]. Our model can accommodate a variable boundary layer thickness parameter, but model outputs of carbon stable isotope discrimination, presented here assume a minimal diffusive boundary layer thickness. Maberly et al. [15] showed that Δ values of this magnitude (>20) from field-collected rhodophytes imply that discrimination by the organism is dominated by RUBISCO carboxylation, not CO_2_ diffusion. Furthermore, [15] hypothesized that the inherently low maximum photosynthetic rates of CO_2_-using red algae may represent an adaptation to low light environments that ensures the capacity to fix carbon is commensurate with low rates of light absorption as a means to avoid photodamage (see also [13]). Similar results showing more negative values of δ^13^C in other macroalgae grown at lower PFD indicate increased isotopic discrimination by RUBISCO [51]. These combined results suggest that geographic range expansions and increased production of CO_2_-using algae under OA may be constrained by a lack of adaptive capacity to exploit the added CO_2_ in seawater. This interpretation is further supported by the model result that CO_2_ had a stronger effect on net photosynthetic rate under light-limited, compared to light saturated, conditions.

### Model outcomes predict the largest climate change effects on macroalgae will be during warming summers and in shallow, well mixed waters

It has been observed that increasing temperature and increasing *p*CO_2_ can have competing effects on the growth and photosynthetic rates of seagrasses (R. Zimmerman, pers. comm.). In a red macroalga with a CCM, high *p*CO_2_ increased the photosynthetic rate at low temperature [22]. Our model predicts that effect for seaweeds without CCMs, but the effect at saturating PFD is much stronger than at limiting PFD. There is a complex interrelationship between the multiple stressors of photosynthetic rate. Predicting the consequences of climate change on productivity requires knowledge of the light environment as well as the pH and temperature, with the greatest effects at high temperatures, and saturating light intensities. While some terrestrial organisms are strongly affected by warming winters (e.g., [52]), our results suggest that the largest effects on macroalgae will be during warming summers and in shallow habitats.

The stressors of photosynthetic rate, examined here, are likely to affect other aspects of algal physiology that contribute to growth and survival under climate change. Thermal effects on rates of photorespiration, respiration in the light were not modeled here. The model includes thermal effects on respiration inherent in the net photosynthetic rate versus temperature relationships used to parameterize the model. However, extrapolation from this model to growth rates would require greater knowledge of the temperature and boundary layer sensitivity of many additional uptake and metabolic processes.

The model presented here serves as a framework upon which principal variables associated with climate change (*p*CO_2_ in, and temperature of, seawater), can be evaluated with respect to their effects on the productivity of phototrophs that depend on diffusive uptake, only, of CO_2_ for photosynthesis. As data accumulate from studies targeting CO_2_-users, comparisons can be made with predictions from the model about the relative importance and magnitudes of effect of these variables on primary production. Based on our understanding of the physiological responses of this group of phototrophs to multiple environmental stressors, we predict that climate change will increase their productivity slightly in the near future and may facilitate range expansions. An accurate understanding is crucial to inform community and ecosystem-level models that seek to predict the extent to which bottom-up effects on production due to changed conditions and resource availabilities will matter ecologically.

## Supporting Information

S1 Appendix Analysis of Diffusion Pathlength Effects. (DOCX)

S2 Appendix Multimodel Inference Method. (DOCX)

S1 Data Predicted values of isotopic composition of plants as a function of seawater temperature and CO_2_ as a fraction of total Carbon. (XLSX)

S1 Table Summary of data sources used for model parameterization. (DOCX)
