# Supplementary material for: Predicting Effects of Ocean Acidification and Warming on Algae Lacking Carbon Concentrating Mechanisms
Source: PLoS One. 2015 Jul 14;10(7):e0132806. doi: 10.1371/journal.pone.0132806 (PMC4501704; doi:10.1371/journal.pone.0132806)
Supplement: S1 Appendix — (DOCX) [file pone.0132806.s001.docx]

**S1 Appendix: Analysis of diffusion pathlength effects**

The relationship between estimated net photosynthetic rate and diffusive boundary layer thickness is described by a negative power function. Both variables were log transformed to linearize the function for analysis with ANCOVA. Two questions about this relationship were of interest: (1) What are the effect sizes of elevated *p*CO_2_ to enhancement of photosynthesis under saturating light? (2) Do the slopes of declining photosynthesis with increasing boundary layer thickness lessen with light intensity?

The effect size due to *p*CO_2_ treatment under saturating light intensities was analyzed across the two saturating light intensities modeled (100 and 400 µmol photons ^.^ m^-2 .^ s^-1^) by pooling those data into one group. Slopes of declining net photosynthesis with increasing boundary layer thickness obtained from a separate slopes model of the ANCOVA for each *p*CO_2_ level were similar and ranged from -0.95 at 380 µatm to -0.91 at 940 µatm. The effect sizes in log transformed units of enhanced net photosynthesis in response to elevated *p*CO_2_ levels relative to that at 380 µatm (i.e., increases in value of the intercept) are shown below in Table A.

**Table A**: **Estimated log effect size of elevated levels of *p*CO_2_ relative to photosynthetic rates measured at 380 µatm**.

| *p*CO_2_ | Effect size |  |
| --- | --- | --- |
| µatm | Estimate | Std. Error |
| 400 | 0.09 | 0.07 |
| 460 | 0.43 | 0.07 |
| 540 | 0.55 | 0.07 |
| 620 | 0.64 | 0.07 |
| 700 | 0.73 | 0.07 |
| 780 | 0.86 | 0.07 |
| 860 | 0.97 | 0.07 |
| 940 | 1.05 | 0.07 |

To address the second question, our main interest was in comparing between light-saturated versus light-limited conditions. We pooled together the two clearly light-saturated intensities (100 and 400 µmol photons ^.^ m^-2 .^ s^-1^) into one group and the two clearly light-limited intensities (10 and 35 µmol photons ^.^ m^-2 .^ s^-1^) into another group. In order to focus on the question of changing slopes of net photosynthesis as a function of boundary layer thickness, we removed the effect of *p*CO_2_ level by expressing the response variable, log(net photosynthetic rate), into standard normal deviates for each *p*CO_2_ level separately. We used these residuals in the homogeneity of slopes model of ANCOVA to test whether the slopes of log(net photosynthesis) as a function of log(boundary layer thickness) for the light-saturated and light-limited groups were the same. We also estimated the percent of variance explained, r^2^, separately for each groups’ regression line.

The slope for the light-saturated group was significantly steeper (i.e., more negative) than that for the limited group (*F*_light*slope(1,2156)_ = 6.22, p=0.01; Table B). The difference in slopes means that the decline in photosynthetic rate with increasing thickness of the boundary layer is greater for light-saturated than for light-limited plants. With respect to model fit, the percentage of variance explained for the regression of log(net photosynthetic rate) on log(boundary layer thickness) for the light-saturated group (r^2^= 0.773) was greater than that for the light-limited group (r^2^=0.677). The better fit of the regression of photosynthesis on boundary layer thickness of the light-saturated group indicates that diffusion strongly limits photosynthesis at both 100 and 400 µmol photons ^.^ m^-2 .^ s^-1^ and across all 9 *p*CO_2_ levels. In contrast, the poorer fit of the light-limited group reflects the transition from diffusion to light limiting photosynthesis in the range of 10-35 µmol photons ^.^ m^-2 .^ s^-1^.

**Table B**: **Slope of estimated log(net photosynthesis) versus log(boundary layer thickness) for light-saturated and light-limited plants**. Value represents estimate of slope ± s.e. and the percent of variance explained by each regression.

| Group | Parameter | Estimate | Std. Error | r^2^ |
| --- | --- | --- | --- | --- |
| Saturated | Slope | -1.264 | 0.021 | 0.773 |
| Limited | Slope | -1.183 | 0.025 | 0.677 |
