# Supplementary material for: Predicting Effects of Ocean Acidification and Warming on Algae Lacking Carbon Concentrating Mechanisms
Source: PLoS One. 2015 Jul 14;10(7):e0132806. doi: 10.1371/journal.pone.0132806 (PMC4501704; doi:10.1371/journal.pone.0132806)
Supplement: S2 Appendix — (DOCX) [file pone.0132806.s002.docx]

**S2 Appendix: Description of Multimodel Inference**

Burnham and Anderson’s [47] approach relies on AIC differences, Akaike weights and evidence ratios to evaluate a group of competing models. We used corrected AIC scores (AICc) since the sample sizes used in experimental studies from which parameter estimates are based were small relative to the number of model parameters estimated. The difference in AICc value, or Δ_i_, represents the corrected Akaike Information Criteria (AICc) for the *i*^th^ model minus the smallest AICc; this is a relative measure and models with Δ_i_ < 2 have substantial support. Akaike weights, *w_i_*, are the relative likelihood that the i^th^ model is the best supported model given the data and the specified group of competing models. By definition, Δ_i_ = 0 for the best-supported model and Σ*w_i_* =1. Evidence ratio is the comparison of two competing models and is *w_i_* / *w_j_*. This gives the relative odds of one model against another. For example, a ratio of six would suggest model *i* is six times more likely to be the best model than model *j*. Ideally, one would like to find a single model for which Δ_i_ < 2 and all evidence ratios are much larger than 1. In practice, usually a subset of models are equally plausible and when this occurred, we used model averaging in which calculation of the average parameter estimate is weighted by *w_i_*.
