# Supplementary material for: Predicting Effects of Ocean Acidification and Warming on Algae Lacking Carbon Concentrating Mechanisms
Source: PLoS One. 2015 Jul 14;10(7):e0132806. doi: 10.1371/journal.pone.0132806 (PMC4501704; doi:10.1371/journal.pone.0132806)
Supplement: S1 Table — (DOCX) [file pone.0132806.s004.docx]

| **S1 Table: Summary of data sources used for model parameterization** | |  |
| --- | --- | --- |
| **Parameter** | **Species** | **Citation** |
| Photosynthetic Rate as a Function of Temperature | *Lomentaria baileyana* | 18 |
|  | *Lomentaria orcadensis* | 18 |
|  |  |  |
| Photosynthetic Rate as a Function of PFD | *Lomentaria articulata* | 12 |
|  | *Delesseria sanguinea* | 12 |
|  | *Lomentaria baileyana* | 18 |
|  | *Lomentaria orcadensis* | 18 |
|  | *Plocamium cartilagineum* | Kübler et al., in prep. |
|  |  |  |
| Photosynthetic Rate as a Function of *p*CO_2_ | *Plocamium cartilagineum* | Kübler et al., in prep. |
|  |  |  |
| Stable Carbon Isotope Discrimination weights (α_c_ vs α_d_) | *Lomentaria articulata* | 15 |
|  | *Delesseria sanguinea* | 15 |
|  |  |  |
| δ^13^C values | *Plocamium cartilagineum* | 15 |
|  | *Lomentaria articulata* | 11, 15, 16 |
|  | *Delesseria sanguinea* | 15 |

The species represented in this table use only CO_2_ from diffusive uptake based on δ^13^C values from replicate field collected specimens [11, 15] and 3-week laboratory acclimation experiments to different CO_2_ and O_2_ concentrations [16]. All photosynthesis data represent short-term measurements (5-10 min.) in well-stirred chambers and are assumed to represent conditions of minimum boundary layer thickness. For each parameter, the species listed were those used to represent the range and pattern of response to the specified environmental variable from data provided in the cited publication from which parameter values were established. Despite their morphological and phylogenetic differences, CO_2_-only using temperate seaweeds show similar area-specific photosynthetic rates to variations in light and temperature.
